# Chemical, Bioactive, and Antioxidant Potential of Twenty Wild Culinary Mushroom Species

**DOI:** 10.1155/2015/346508

**Published:** 2015-06-25

**Authors:** S. K. Sharma, N. Gautam

**Affiliations:** ^1^Department of Plant Pathology, CSK, Himachal Pradesh Agriculture University, Palampur 176 062, India; ^2^Centre for Environmental Science and Technology, School of Environment and Earth Sciences, Central University of Punjab, Bathinda 151 001, India

## Abstract

The chemical, bioactive, and antioxidant potential of twenty wild culinary mushroom species being consumed by the people of northern Himalayan regions has been evaluated for the first time in the present study. Nutrients analyzed include protein, crude fat, fibres, carbohydrates, and monosaccharides. Besides, preliminary study on the detection of toxic compounds was done on these species. Bioactive compounds evaluated are fatty acids, amino acids, tocopherol content, carotenoids (*β*-carotene, lycopene), flavonoids, ascorbic acid, and anthocyanidins. Fruitbodies extract of all the species was tested for different types of antioxidant assays. Although differences were observed in the net values of individual species all the species were found to be rich in protein, and carbohydrates and low in fat. Glucose was found to be the major monosaccharide. Predominance of UFA (65–70%) over SFA (30–35%) was observed in all the species with considerable amounts of other bioactive compounds. All the species showed higher effectiveness for antioxidant capacities.

## 1. Introduction

Wild mushrooms have long been considered as highly nutritious tasty food items from ancient time [1–3]. Besides nutritional importance wild edible mushrooms are now well known for their pharmaceutical constituents [4, 5]. Presently, there are several mushroom species which have established therapeutic properties [6–11]. In addition, mushroom extract is considered as important remedies for the prevention and treatment of many diseases for thousands of years in several parts of the world [12, 13]. Mushrooms are known to contain immunomodulating compounds which help to improve immune function in cancer patients during radio and chemotherapy and help to prolong survival times in some types of cancer [14]. Another aspect of mushrooms is lowering blood pressure and free cholesterol in plasma [15]. Major bioactive compounds extracted from mushrooms are well known for their antioxidant [16], antitumor, and antimicrobial properties [17]. The nutritive nutraceuticals present in mushrooms are dietary fibres, polyunsaturated fatty acids (PUFA), proteins, amino acids, keto acids, minerals, antioxidative vitamins, and other antioxidants [18–20]. Currently 14,000 mushroom species are known to exist. Out of these, about 50% species are reported to possess varying degrees of edibility and almost 3000 species spread over 31 genera are regarded as prime edible mushrooms. To date only 200 of them are experimentally grown, 100 of them are economically cultivated, approximately 60 are commercially cultivated, and about 10 have reached industrial scale production in many countries [21]. Furthermore, about 2000 are medicinal mushrooms with variety of health benefits and 270 species are now considered as potential therapeutic or preventative agents that are ensured for human health perspective. The poisonous mushrooms are relatively small in number (approximately 1%) but there is an estimate that about 10% may have poisonous attributes while 30 species are considered to be lethal [21].

The northern Himalayan regions of India include the states of Himachal Pradesh (30°22′ to 33°12′N latitude and 75°45′ to 79°04′E longitude), Uttarakhand (28°43′N to 31°28′N latitude and 77°34′E to 81°03′E longitude), and some parts of Jammu and Kashmir (34°8′N and 77°34′E). The regions in these states have extensive areas under forest cover (i.e., more than 50%). The people of these areas use different mushroom species for culinary purposes. They have very little idea about the medicinal importance of these mushroom species. Their methods of identification are primarily based upon sweet smell and color. Further, the knowledge about the edible species is restricted to the old aged people. Documentations studies of these edible species have been done by several workers [22] but the studies on chemical composition and medicinal importance are still lacking besides the presence of toxic compounds in them. In view of this, under present investigations twenty of such wild culinary mushroom species have been evaluated for their chemical, bioactive, and antioxidant potential.

## 2. Materials and Methods

### 2.1. Collection and Processing of Samples

All the samples were collected during the frequent surveys to the different regions of northern Himalayas (Table 1). After this, the samples were vacuum-dried and preserved in air-tight cellophane bags, with a small amount of 1-4-paradichlorobenzene in porous packets to keep them free of insects, for further analysis.

### 2.2. Chemical Evaluation

Samples were powdered and evaluated for protein, fat, carbohydrates, ash, and crude fibres. Crude protein content was estimated using the Kjeldahl method by calculating total nitrogen (N) and protein content was expressed by N × 4.38 [23]. Crude fat was estimated using a Soxhlet apparatus by extraction of known weight of powered samples with petroleum ether. Ash content was calculated by incineration in silica dishes at 525 ± 20°C containing 5–10 g/sample. Fibres content was estimated on a fat-free sample using the acid-alkali method (1.25% each). Total carbohydrates percentage was calculated by the difference as the total weight − (moisture content + protein content + crude fat + ash content + crude fibres).

For toxic metal detection, diluted HCl (2%) and copper foil (1 × 1/2 cm strip, purified or pretreated with concentrated HNO_3_ or diluted 3 HCl and rinsed in distilled water) were taken. After that samples (powered samples) were acidified with 10–20 mL of 5 diluted HCl (2%) until colour changed from fairy pink to litmus. Then strips of copper foil were added and boiled for 30 minutes with addition of water from time to time to replace the losses by evaporation. The heavy metals got deposited on the copper foil and gave characteristics color to it. The color deposited on the Cu foil was noted after 30 minutes and results were interpreted for the presence of heavy metals [24].

For monosaccharide composition, samples (0.1 g) were extracted with 2.5 mL and 1.5 mL and finally with 1 mL of 70% aqueous methanol. After this, the extract was centrifuged at 4000 rpm (4°C) for 10 min. Supernatant was collected and volume was made up to 5 mL with 70% methanol. The extract was passed through Millipore filter (0.45 *μ*m) and injected to the HPLC [24].

### 2.3. Bioactive Evaluation

#### 2.3.1. Fatty Acid Composition

Powdered samples were dissolved in 1 mL of solution (prepared by sodium hydroxide pellets (45 g) in 300 mL of 50% methanol and vortexing for 1 minute; then the solution was left for 5 minutes at 100°C, vortexed again for 1 minute, and left at 100°C in a water bath for 25 minutes). Methylation was done by adding 2 mL of solution (6N hydrogen chloride in methanol) and then vortexed for 1 minute, followed by heating (80°C) in a water bath. For extraction of fatty acids, 1.25 mL of solution (25 mL methyl ter-butyl ether added to hexane) was added, and the solution was shaken for 10 minutes followed by removal of upper layer and addition of 3 mL of solution (10% sodium hydroxide in water while stirring). Finally, the top phase (2/3) was removed and transferred into a gas chromatography vial and injected. Unsaturation index (UI) was calculated as (mol% of each (poly)unsaturated fatty acid × number double bonds per each fatty acid)/100.

#### 2.3.2. Amino Acids

Powdered samples (0.1 g) were extracted with 2.5 mL followed by 1.5 mL and 1 mL of 70% aqueous methanol. After this, centrifugation was done for 10 minutes (4000 rpm) at 4°C. Supernatants were dissolved in aqueous methanol and the volume was made up to 5 mL. It was now passed through Millipore filter (0.45 *μ*m). After this, samples (10 *μ*L) were dried using vacuum oven and, to these dried samples, 20 *μ*L derivatising agent (prepared by ethanol : triethylamine : water : phenylisothiocaynate) was mixed with it and redried it. Now the samples were left for 25 minutes at room temperature. Lastly, 1 mL ethanol was added and injected into UPLC (Waters India Pvt. Ltd.).

#### 2.3.3. Tocopherol Composition

Tocopherol composition was estimated following standard method [25]. For this, samples were mixed with butylated hydroxytoluene (BHT) in hexane (10 mg/mL; 100 *μμ*L) and IS solution in hexane (*δ* tocopherol; 1.6 *μ*g/mL; 250 *μ*L). Thereafter, samples (500 mg) were vortexed for 1 min with methanol (4 mL). Then samples were again vortexed with hexane (4 mL). After this, 2 mL of saturated NaCl aqueous solution was added, and the mixture was vortexed (1 min), followed by centrifugation at 4000 g for 5 min and the upper layer was separated. The samples were again reextracted twice with hexane. The extracts were then vacuum-dried and redissolved in hexane (1 mL), followed by dehydration with anhydrous sodium sulphate, then filtered and transferred into a dark injection vial, and analysed by HPLC (Waters India Pvt. Ltd.). Chromatographic comparisons were made by authentic standards. Tocopherol contents in mushroom samples were expressed in *μ*g per g of dry mushroom.

#### 2.3.4. Evaluation of Other Bioactive Compounds

For *β*-carotene and lycopene estimation, dried powdered samples (~5 g) were extracted with 100 mL of methanol at 25°C (150 rpm) for 24 hours and filtered through Whatman Number 4 filter paper. The residue was again reextracted with 2 additional 100 mL portions of methanol. These extracts were evaporated to dryness at 42°C, then redissolved in methanol at a concentration of 50 mg/mL, and stored at 4°C. The dried methanolic extract (100 mg) was shaken vigorously with 10 mL of acetone/hexane mixture (4 : 6) for 1 minute and filtered. The absorbance of the filtrate was measured at 453, 505, and 663 nm [26]. *β*-carotene and lycopene content were estimated using the following equation:

(1) Lycopene mg/100 mL=0.0458×A663+0.372×A505−0.0806×A453β-carotene mg/100 mL=0.216×A663−0.304×A505+0.452×A453.

For phenolic compounds quantification, powdered samples (1 mL) were mixed with Folin and Ciocalteu's phenol reagent (1 mL). After 3 minutes, 1 mL of saturated sodium carbonate solution was added to the mixture, and the volume was adjusted to 10 mL with distilled water. The reaction was kept in the dark for 90 minutes, after which the absorbance was read at 725 nm. Gallic acid was used to calculate the standard curve (0.01–0.4 mM; *R* ^2^ = 0.9999) and the results were expressed as milligrams of gallic acid equivalents per gram of extract [27].

Total flavonoids of the sample extracts were measured by AlCl_3_ method [28]. For this, an aqueous extract (1.5 mL) was mixed with deionized distilled water (5 mL) and 0.3 mL of 5% NaNO_2_. After five minutes of incubation at room temperature, 1.5 mL of 2% aluminium trichloride (AlCl_3_) solution was added. After the next 6 minutes 2 mL of 1 M NaOH was added. The mixture was vigorously shaken on orbital shaker for 5 min at 200 rpm and the absorbance was read at 510 nm against a blank. Quercetin with different concentrations was used as a standard.

For ascorbic acid quantification, standard ascorbic acid solution (5 mL L-ascorbic acid in 3% phosphoric acid) was added to 5 mL of phosphoric acid. A microburette was filled with dye, and the samples were titrated with the dye solution to a pink color, which persisted for 15 seconds. The dye factor (milligrams of ascorbic acid per milliliter of dye using formula: 0.5/titrate) was determined. A sample was prepared by taking 10 g of sample grounded in metaphosphoric acid, and the volume was increased up to 100 mL. It was titrated after filtration until a pink color appeared [24]. The amount of ascorbic acid was calculated with the use of the following equation:

(2) mg of ascorbic acid per 100 g  or mL=Titrate×Dye factor×Vol. madeAliquot of extract×wt. of sample×100.

Anthocyanidins were quantified by using standard method [29]. Briefly, 0.5 g of samples was mixed with the solvent (mixture of 85 : 15 (v/v) of ethyl alcohol and hydrochloric acid 1.5 M) followed by ultrasonication for 15 minutes and filtration through Whatman filter paper number 1. Standard solution was prepared with cyaniding chloride with a concentration of 5–15 *μ*g/mL, in solvent which was used. The absorption was measured at 546 nm. The total quantity of anthocyanins (expressed in g of cyaniding chloride/100 g extract) = (*A*_*p*_ × *m*_st_ × *f* × 100)/(*A*_st_ × *m*_*p*_), where *A*_*p*_ is absorption rate of the sample solution; *m*_*p*_ is mass of the processed sample, in g; *A*_st_ is absorption rate of the standard solution; *m*_st_ is mass of the processed standard solution, in g; and *f* is dilution coefficient.

### 2.4. Antioxidant Assays

DPPH scavenging activity was measured with adding DPPH (200 *μ*L) solution at different concentrations (2–10 mg/mL) to 0.05 mL of the samples (dissolved in ethanol). An equal amount of ethanol was added to the control. Ascorbic acid was used as the control [30]. The absorbance was read after 20 min at 517 nm and the inhibition was calculated using the formula

(3) $$\begin{array}{c} \text{DPPH scavenging effect }\left(\;\%\;\right)=\frac{A_{0}-A_{P}}{A_{0}}\times 100, \end{array}$$

where *A*_0_ was the absorbance of the control and *A*_*P*_ was the absorbance in the presence of the sample.

For ABTS radical scavenging activity 10 *μ*L of the sample was added to 4 mL of the diluted ABTS^•+^ solution (prepared by adding 7 mM of the ABTS stock solution to 2.45 mM potassium persulfate, kept in the dark, at room temperature, for 12–16 h before use). The solution was then diluted with 5 mM phosphate-buffered saline (pH 7.4) and absorbance was measured at 730 nm after 30 min [31]. The ABTS radical scavenging activity was calculated as

(4) $$\begin{array}{c} S\%=\left(\;\frac{A_{\text{control}}-A_{\text{sample}}}{A_{\text{control}}}\;\right)\times 100. \end{array}$$

For reducing power estimation, samples (200 *μ*L) were mixed with sodium phosphate buffer (pH 6.6), 1 mM FeSO_4_, and 1% potassium ferricyanide and incubated for 20 min at 50°C after that trichloroacetic acid was added and the mixtures were centrifuged. Supernatant (2.5 mL) was mixed with an equal volume of water and 0.5 mL 0.1% FeCl_3_. The absorbance was measured at 700 nm [32].

For Fe^2+^ chelating activity, 1 mL of the sample (2–10 mg/mL) was mixed with 3.7 mL of ultrapure water, after that the mixture was reacted with ferrous chloride (2 mmol/L, 0.1 mL) and ferrozine (5 mmol/L, 0.2 mL) for 20 min and the absorbance was read at 562 nm with using EDTA as control. The chelating activity was calculated using the formula

(5) $$\begin{array}{c} \text{chelating activity }\left(\;\%\;\right)=\left[\;\frac{\left(A_{b}-A_{s}\right)}{A_{b}}\;\right]\times 100, \end{array}$$

where *A*_*b*_ is the absorbance of the blank and *A*_*s*_ is the absorbance in the presence of the extract [33].

The scavenging activity of superoxide anion radicals was measured following standard method [34]. Samples (0–2.0 mg/mL, 1 mL) and Tris-HCl buffer (50.0 mM, pH 8.2, 3 mL) were incubated in a water bath at 25°C for 20 min and after this pyrogallic acid (5.0 mM, 0.4 mL) was added. HCl solution (8.0 M, 0.1 mL) was added to terminate the reaction after 4 min. The absorbance of the mixture was measured at 320 nm. The scavenging ability was calculated using the following formula:

(6) $$\begin{array}{c} \text{scavenging ability }\left(\;\%\;\right)=\left(\;1-\frac{A_{\text{sample}}}{A_{\text{control}}}\;\right)\times 100, \end{array}$$

where *A*_control_ is the absorbance of control without the polysaccharide sample and *A*_sample_ is the absorbance in the presence of the polysaccharide sample.

For ferric reducing antioxidant power (FRAP) assay, firstly, FRAP reagent was prepared by mixing TPTZ (2.5 mL, 10 mM in 40 mM HCl), 25 mL of 300 mM acetate buffer, and 2.5 mL of FeCl_3_·6H_2_O. After this, freshly prepared FRAP reagent (1.8 mL) was taken in a test tube and incubated at 30°C in water bath for 10 minutes. Then, absorbance was read at 0 min (*t*_0_). After this, 100 *μ*L of sample extract or standard and 100 *μ*L of distilled water were added to the test tube, mixed, and incubated at 30°C for 30 minutes. Then, the absorbance was taken at 593 nm (*t*_30_). Ferrous sulphate was used as standard [35, 36]. FRAP activity was determined against a standard curve of ferrous sulphate and the values were expressed as *μ*M Fe^2+^ equivalents per gram of extract and calculated using the following equation:

(7) FRAP value=Absorbance sample+FRAP reagent−Absorbance FRAP reagent.

### 2.5. Statistical Analysis

All experiments were performed 3 times and with 3 replicates. The results were analyzed using one-way analysis of variance (ANOVA). *p* < 0.05 was considered significant, and SPSS software (SPSS Inc., Chicago, IL, USA) was used to calculate differences. Tukey-HSD at *p* < 0.05 test was used to determine significant differences.

## 3. Results 

### 3.1. Chemical Evaluation

Nutrient composition of the wild culinary mushrooms is shown in Table 2. Protein was found in high levels and varied between 5.16% in* Inocybe splendens* and 22.63% in* Agaricus arvensis*. Protein percentage in* Pleurotus cystidiosus* (20.69%),* Amanita caesarea* (19.72%),* Agaricus campestris* (18.38%),* Cantharellus cibarius* (18.19%), and* Lentinus cladopus* (18.59%) was also found to be high. All the twenty culinary species were found to be low in fat content. Fat ranged from 0.10% in* Laccaria laccata* to 0.38% in* Inocybe splendens*. In general these wild culinary mushrooms consumed by local people were found to be higher in protein and low in fat although differences were observed in net value of individual species. Crude fibres ranged from 1.08% in* Hygrocybe coccinea* to 2.42% in* Lentinus cladopus*. Ash content varied between 0.11% in* Inocybe splendens* and 0.96% in* Agaricus arvensis*. Carbohydrates, calculated by difference, were also found to be in abundant amounts and their percentage was ranged from 31.19% in* Inocybe splendens* to 57.12% in* Agaricus arvensis*. Nutrient contents of* Inocybe splendens, Hygrocybe nivea*, and* Conocybe tenera* were found to be less as compared to other species.

There are several reports about the toxicity reports due to mushrooms on humans in these areas and hence preliminary studies were done to check the toxicity level of mushrooms. For all the twenty species being used by the people for culinary purposes the test was found to be negative. That means these species are nontoxic and hence recommended for consumption.

All the culinary mushroom species contained glucose and rhamnose as the principal carbohydrates (Table 3). Nevertheless, the present study also describes the presence of xylose and mannose in all the studied species. However, galactose and fructose were detected in very low percentage in some of the species.* Russula mairei* contained lowest percentage of glucose (21.60%) and* Agaricus arvensis* contained highest percentage of glucose (64.12%).

### 3.2. Bioactive Evaluation

The results of fatty acid composition (total saturated fatty acids SFA, monounsaturated fatty acids MUFA, and polyunsaturated fatty acids PUFA) of all the species are shown in Table 4. In general, the major fatty acids found in the studied species were linoleic acid (C18:2), followed by oleic acid (C18:1) and palmitic acid (C16:0). Besides these three main fatty acids already described, six more were identified and quantified. PUFA were the main group of fatty acids documented in all the species.* Agaricus arvensis, Amanita caesarea*,* Cantharellus cibarius, Lentinus cladopus*, and* Pleurotus cystidiosus* contained lower value of MUFA but higher percentage of PUFA as compared to other species due to the higher percentage of linoleic acid. However, UFA predominated over SFA in all the studied species ranging from 65 to 70%.

Amino acid composition of all the species is shown in Table 5. In all the species aspartic acid (0.19–0.39%) was found to be predominated amino acid followed by tyrosine (0.10–0.21%), arginine (0.12–0.29%), alanine (0.04–0.14%), and proline (0.01–0.07%).* Amanita caesarea, Agaricus arvensis, Cantharellus cibarius, Lentinus cladopus, and Pleurotus cystidiosus* contained maximum amount of these amino acids. Tocopherol contents in all the studied mushroom species including three wild are detailed in Table 6. *α*-tocopherol and *β*-tocopherol were found to be present in all the species. However, *γ*-tocopherol was documented from few species only. Tocopherol content was ranged from 0.90 to 4.33 *μ*g/g in all the species.* Cantharellus cibarius* (4.33 ± 0.0 *μ*g/g) contained all the three isomers in higher amount as compared to other species. *β*-tocopherol was found in higher amounts as compared to *α*-tocopherol. *γ*-tocopherol was detected only in nine species.* Cantharellus cibarius* (4.33 ± 0.0 *μ*g/g) contained higher amounts of *γ* tocopherol.

Results obtained for *β*-carotene, lycopene, flavonoids, ascorbic acid, and anthocyanidins composition of all the twenty species are presented in Table 7. Phenols were the major antioxidant component detected in significant amounts from all the species (19.12–63.36 mg/g), followed by anthocyanidins (6.14–14.25 mg cyanidin chloride/100 g extract), flavonoids (1.14–4.17 mg/g) ascorbic acid which was found in small amounts (0.20–0.99 mg/g), *β*-carotene (0.21–0.79 *μ*g/100 g), and lycopene (0.19–0.38 *μ*g/100 g). Each species differed with other species in net amounts of all these components. Species like* Agaricus arvensis, Amanita caesarea, Gymnopilus junonius, Lentinus cladopus*, and* Pleurotus cystidiosus* contained higher values of these components as compared to other species.

### 3.3. Antioxidant Evaluation

Antioxidant properties of all the species were expressed as EC_50_ values for comparison (Table 8). Higher EC_50_ values indicate lower effectiveness in antioxidant properties. EC_50_ values obtained for DPPH radical scavenging activity in all the species showed differences in effectiveness in antioxidant properties. Among all the species* Cantharellus cibarius* showed lowest EC_50_ values (1.76 ± 0.2 mg/mL) followed by* Amanita caesarea* (2.02 ± 0.2 mg/mL) and* Agaricus arvensis* (2.12 ± 0.4 mg/mL). Other species showed slightly higher EC_50_ values and therefore lesser DPPH radical scavenging activity.* Cantharellus cibarius* showed higher DPPH radical scavenging activity and* Inocybe splendens* showed lower DPPH radical scavenging activity than other species.

For ABTS radical scavenging activities EC_50_ ranged from 4.26 to 1.45 mg/mL. Lowest EC_50_ values were obtained for* Amanita caesarea* (1.45 ± 0.6 mg/mL) showing high antioxidant activities of this species. Higher EC_50_ values were obtained for* Hygrocybe nivea* (4.26 ± 0.5 mg/mL) showing lowest ABTS radical scavenging activities of this species. Reducing power of* Cantharellus cibarius* (1.62 ± 0.5 mg/mL) was measured higher than other species. Higher EC_50_ values for reducing activity were measured in* Inocybe splendens* (5.19 ± 0.1 mg/mL).

Higher effectiveness in ferrous ion chelating activity was detected in* Agaricus arvensis* (1.14 ± 0.1 mg/mL) and low effectiveness was detected in* Russula mairei* (2.95 ± 0.3 mg/mL). Nevertheless,* Cantharellus cibarius* (1.27 ± 0.2 mg/mL),* Amanita caesarea* (1.40 ± 0.4 mg/mL),* Hygrocybe coccinea* (1.35 ± 0.2 mg/mL), and* H. nivea* (1.45 ± 0.6 mg/mL) showed lower EC_50_ values than remaining species. EC_50_ values of scavenging ability on superoxide radical were found to be maximum in* Amanita caesarea* (0.44 ± 0.3 mg/mL) and minimum in* Lactarius pubescens* (2.21 ± 0.6 mg/mL).* Gymnopilus junonius* showed maximum FRAP activity with least EC_50_ values (0.78 mg/mL) and* Russula mairei* showed minimum antioxidant activity with high EC_50_ values (2.16 mg/mL).

## 4. Discussion

Although there are previous reports on documentation of culinary edible species from the regions native to northern Himalayas but there are no reports on the evaluation studies as well as toxicity status of all these culinary species. The wild edible species* Agaricus bisporus, Boletus edulis, Morchella esculenta, Cordyceps sinensis*, and* Lentinula edodes* which have been extensively worked out in India and other parts of the world for their compositional and medicinal aspects have not been undertaken for investigations presently [37–40]. Compositional studies showed that most of the culinary species were rich in protein, carbohydrates, and low in fat. There are several reports on richness of wild edible mushrooms with protein and carbohydrate contents and low fat levels which directly make them nutritionally rich [24, 25, 41]. Nevertheless, under present studies the differences between the nutrient concentrations of all the species differed but* Agaricus arvensis*,* Pleurotus cystidiosus, Amanita caesarea, Agaricus campestris, Cantharellus cibarius*, and* Lentinus cladopus* showed higher nutrient percentage which is comparable to other wild and commercially cultivated species [25, 42]. The crude fat content detected in all the species was not found to be significantly different. Crude fibres were detected in appreciable percentage from all the species which make them important in nutritional point of view. The results are in conformity with the previous reports on several wild edible* Pleurotus* and* Lentinus* species from northwest India [24, 43]. The species* Inocybe splendens, Hygrocybe nivea*, and* Conocybe tenera* were not found to contain higher percentage of nutrients. Although previous reports showed that nutrients composition in wild species is less as compared to cultivated species [25], however* Agaricus arvensis*,* Pleurotus cystidiosus, Amanita caesarea, Agaricus campestris, Cantharellus cibarius*, and* Lentinus cladopus* were found to be rich in protein and carbohydrates similar to commercially grown species [25].

Fatty acid composition showed the dominance of UFA over SFA in all the studied mushrooms species, which is in conformity with the other studies [41]. The differences were observed in net amounts in all the species. Unsaturation index of* Agaricus arvensis* and* Lentinus cladopus* (0.57 ± 0.02%) was found to be significantly higher than other species Whereas,* Inocybe splendens* (0.05 ± 0.0%) showed least Unsaturation Index. High UFA shows the medicinal importance of these culinary mushrooms as these increase the HDL cholesterol and decrease LDL cholesterol, triacylglycerol, lipid oxidation, and LDL susceptibility to oxidation [44]. Predominance of UFA over SFA in all the species shows similar results as obtained for other wild and commercially cultivated species [24, 25]. *α*- and *β*-tocopherol were detected in higher amounts than third isomer in all the studied species. Similar findings were made in other wild and cultivated species with higher *α*- and *β*-tocopherol than *γ*-tocopherol [25]. The high levels of these two compounds correspond with a higher oxidative activity, which is associated with cardiovascular protection [45]. Phenolic compounds were detected in higher amounts than other bioactive compounds. Presence of high phenolic compounds accounts for the high antioxidant properties of all the species [42]. *β*-carotene, lycopene, and ascorbic acids were detected in low amounts. Anthocyanidins were also detected from these wild species in appreciable amounts. The presence of these functional medicinal compounds in medicinal and/or edible mushroom is due to habitat or substrates in which these grow to be high in the functional molecules. The categories of these molecules are anthocyanidins, beta-glucans, selenium, ganoderic acid, triterpenes, or cordycepin. The compounds identified in these extracts show that at least a part of the functional compounds in medicinal and/or edible mushroom is due to growing mushrooms on substrates that are high in the functional molecules. To these categories can be added anthocyanidins, beta-glucans, selenium, ganoderic acid, triterpenes, or cordycepin. The amounts of these have been found to vary with the type of extraction as ethanolic extract yields higher amounts of anthocyanidins as compared to methanolic, hot water, and cold water extracts [29].

All the studied species showed significant antioxidant properties measured on the basis of EC_50_ values. Nevertheless, each species showed different antioxidant activity with highly effective and less effective EC_50_ values. Better antioxidant properties of some species are due to presence of higher phenolic compounds, *β*-carotene, lycopene, ascorbic acids, anthocyanidins, and tocopherol amounts in them. High reducing power of some species is due to the presence of higher amounts of reducers (antioxidants) in them. Presently investigated species are commonly used for culinary purposes in the regions native to northern Himalayas. Many of the species in these regions are not evaluated previously for detailed compositional analysis. Their knowledge is restricted to old aged villagers of the regions and neglected for the commercial exploitations. There are no positive reports on toxicity of these mushrooms analyzed presently; hence these are safe for further experimental work related to drug discovery. All the culinary species contained important and useful nutraceuticals such as unsaturated fatty acids, phenolics, carotenoids, ascorbic acid, tocopherols, and anthocyanidins; besides these, some important amino acids were detected in these mushrooms which could be used for the purpose of being used as functional ingredients. Since nutraceuticals are powerful in maintaining and promoting health, longevity, and life quality, the commercial exploitation of these species will certainly create an impact on nutritional therapy and also will be beneficial today's food industry. Direct use of these species for consumption and other culinary aspects is safe and health promoting with advantage of the additive effects of all the bioactive and antioxidant compounds present in these species.

## Figures and Tables

**Table 1:** List of wild culinary species collected from different localities of twenty species from northern Himalayas.

Species	Collection locality	Altitude (meters)
*Agaricus arvensis *	Shimla (Himachal Pradesh)	2300
*A. campestris *	Kullu (Himachal Pradesh)	2200
*A*. *comtulus *	Jhatingri (Himachal Pradesh)	2300
*A. silvicola *	Dharamshala (Himachal Pradesh)	1800
*Amanita caesarea *	Janjehli (Himachal Pradesh)	2500
*A. citrine *	Mcleodganj (Himachal Pradesh)	2000
*A. fulva *	Mcleodganj (Himachal Pradesh)	2000
*Cantharellus cibarius *	Khajjiar (Himachal Pradesh)	2400
*Conocybe tenera *	Kufri (Himachal Pradesh)	2400
*Gymnopilus junonius *	Nanital (Uttarakhand)	2300
*Hygrocybe coccinea *	Jhatingri (Himachal Pradesh)	2300
*H. nivea *	Dharamshala (Himachal Pradesh)	1800
*Inocybe splendens *	Sonamarg (Jammu and Kashmir)	2800
*Lactarius pubescens *	Mcleodganj (Himachal Pradesh)	2000
*Laccaria laccata *	Mcleodganj (Himachal Pradesh)	2000
*Lepista nuda *	Khajjiar (Himachal Pradesh)	2400
*Lentinus cladopus *	Bhadrol (Himachal Pradesh)	1200
*Pleurotus cystidiosus *	Palampur (Himachal Pradesh)	1400
*Russula lepida *	Mcleodganj (Himachal Pradesh)	2000
*R. mairei *	Mcleodganj (Himachal Pradesh)	2000

**Table 2:** Percent chemical composition of twenty wild culinary mushroom species collected from northern Himalayan regions.

Species	Protein	Crude fat	Fibres	Ash	Carbohydrates
*Agaricus arvensis *	22.63 ± 0.2^l^	0.22 ± 0.0^a^	2.11 ± 0.0^c^	0.96 ± 0.0^a^	57.12 ± 2.7^m^
*A. campestris *	18.38 ± 0.6^j^	0.12 ± 0.0^a^	1.17 ± 0.0^b^	0.87 ± 0.0^a^	43.45 ± 2.4^j^
*A. comtulus *	12.85 ± 0.2^f^	0.20 ± 0.0^a^	1.93 ± 0.0^b^	0.30 ± 0.0^a^	39.28 ± 3.8^i^
*A. silvicola *	17.14 ± 0.1^i^	0.26 ± 0.0^a^	1.82 ± 0.0^b^	0.54 ± 0.0^a^	48.53 ± 3.2^k^
*Amanita caesarea *	19.72 ± 0.3^h^	0.21 ± 0.0^a^	1.18 ± 0.0^b^	0.42 ± 0.0^a^	42.16 ± 1.9^l^
*A. citrine *	11.12 ± 0.1^e^	0.17 ± 0.0^a^	1.62 ± 0.0^b^	0.40 ± 0.0^a^	27.15 ± 3.2^a^
*A. fulva *	10.11 ± 0.1^d^	0.22 ± 0.0^a^	1.83 ± 0.0^b^	0.11 ± 0.0^a^	32.12 ± 2.3^d^
*Cantharellus cibarius *	18.19 ± 0.5^j^	0.27 ± 0.0^a^	2.08 ± 0.0^c^	0.55 ± 0.0^a^	56.42 ± 2.9^m^
*Conocybe tenera *	8.61 ± 0.3^c^	0.25 ± 0.0^a^	0.91 ± 0.0^a^	0.26 ± 0.0^a^	37.10 ± 3.7^h^
*Gymnopilus junonius *	14.23 ± 0.1^g^	0.23 ± 0.0^a^	1.23 ± 0.0^b^	0.41 ± 0.0^a^	49.29 ± 7.3^k^
*Hygrocybe coccinea *	12.15 ± 0.6^f^	0.34 ± 0.0^a^	1.08 ± 0.0^b^	0.20 ± 0.0^a^	36.44 ± 2.3^g^
*Hygrocybe nivea *	7.60 ± 0.2^b^	0.26 ± 0.0^a^	1.14 ± 0.0^b^	0.16 ± 0.0^a^	32.14 ± 2.3^d^
*Inocybe splendens *	5.16 ± 0.4^a^	0.38 ± 0.0^a^	0.85 ± 0.0^a^	0.11 ± 0.0^a^	31.19 ± 2.5^c^
*Lactarius pubescens *	15.12 ± 0.7^h^	0.24 ± 0.0^a^	1.98 ± 0.0^b^	0.44 ± 0.0^a^	32.40 ± 2.0^d^
*Laccaria laccata *	10.14 ± 0.4^d^	0.10 ± 0.0^a^	1.95 ± 0.0^b^	0.36 ± 0.0^a^	30.18 ± 4.2^b^
*Lepista nuda *	10.18 ± 0.7^d^	0.25 ± 0.0^a^	1.26 ± 0.0^b^	0.21 ± 0.0^a^	33.13 ± 5.2^e^
*Lentinus cladopus *	18.59 ± 0.9^j^	0.24 ± 0.0^a^	2.42 ± 0.0^c^	0.49 ± 0.0^a^	56.49 ± 2.1^l^
*Pleurotus cystidiosus *	20.69 ± 0.3^k^	0.20 ± 0.0^a^	2.16 ± 0.0^c^	0.42 ± 0.0^a^	52.20 ± 3.6^n^
*Russula lepida *	12.10 ± 0.4^f^	0.28 ± 0.0^a^	1.19 ± 0.0^b^	0.17 ± 0.0^a^	34.15 ± 4.3^f^
*R. mairei *	11.03 ± 0.9^e^	0.22 ± 0.0^a^	1.38 ± 0.0^b^	0.13 ± 0.0^a^	36.40 ± 3.1^g^

Values are expressed as mean ± SE and different letters represent the significant difference in each column with *p* ≤ 0.05 according to Tukey's test.

**Table 3:** Percent monosaccharide composition of twenty species collected from northern Himalayan regions showing richness in glucose, rhamnose, mannose, xylose, and galactose and fructose in lower percentage.

Species	Xylose	Glucose	Rhamnose	Mannose	Galactose	Fructose
*Agaricus arvensis *	10.17 ± 0.3^g^	64.12 ± 2.7^o^	22.10 ± 2.7^k^	4.15 ± 0.7^e^	0.9 ± 0.0^b^	0.06 ± 0.0^a^
*A. campestris *	5.20 ± 0.2^d^	48.63 ± 2.2^k^	17.19 ± 1.5^h^	3.10 ± 0.2^d^	0.7 ± 0.0^b^	0.02 ± 0.0^a^
*A*. *comtulus *	4.21 ± 0.5^c^	35.10 ± 3.0^g^	10.18 ± 1.7^b^	2.32 ± 0.2^c^	0.1 ± 0.0^b^	0.05 ± 0.0^a^
*A. silvicola *	6.46 ± 0.1^e^	44.12 ± 3.7^j^	14.29 ± 1.5^f^	3.19 ± 0.6^d^	0.2 ± 0.0^b^	0.06 ± 0.0^a^
*Amanita caesarea *	12.15 ± 0.4^h^	53.60 ± 2.6^r^	23.17 ± 1.2^l^	5.31 ± 0.6^f^	0.7 ± 0.0^b^	0.01 ± 0.0^a^
*A. citrine *	4.16 ± 0.1^c^	28.60 ± 2.1^d^	12.81 ± 1.6^j^	1.12 ± 0.0^b^	0.3 ± 0.0^b^	ND
*A. fulva *	6.21 ± 0.2^e^	32.15 ± 2.9^f^	14.25 ± 1.7^f^	2.80 ± 0.5^c^	0.2 ± 0.0^b^	ND
*Cantharellus cibarius *	13.27 ± 0.5^i^	59.72 ± 3.4^m^	22.17 ± 2.3^k^	4.98 ± 0.7^e^	0.8 ± 0.0^b^	0.06 ± 0.0^a^
*Conocybe tenera *	2.25 ± 0.1^a^	38.67 ± 2.8^h^	10.29 ± 3.5^a^	1.13 ± 0.0^b^	ND	ND
*Gymnopilus junonius *	8.21 ± 0.5^h^	55.16 ± 3.4^l^	16.18 ± 2.1^g^	2.32 ± 0.2^c^	0.5 ± 0.0^b^	0.05 ± 0.0^a^
*Hygrocybe coccinea *	3.16 ± 0.1^b^	29.16 ± 3.7^e^	11.12 ± 1.5^c^	1.10 ± 0.6^b^	ND	ND
*H. nivea *	3.15 ± 0.0^b^	22.61 ± 2.6^b^	13.16 ± 1.2^e^	1.30 ± 0.6^b^	ND	ND
*Inocybe splendens *	4.29 ± 0.2^c^	28.62 ± 2.5^d^	9.81 ± 1.6^a^	0.98 ± 0.0^a^	ND	ND
*Lactarius pubescens *	5.28 ± 0.3^d^	32.15 ± 2.2^f^	12.25 ± 2.0^d^	2.84 ± 0.7^c^	0.7 ± 0.0^b^	0.02 ± 0.0^a^
*Laccaria laccata *	5.23 ± 0.5^d^	24.12 ± 4.5^c^	12.19 ± 2.1^d^	1.98 ± 0.5^b^	0.2 ± 0.0^b^	0.01 ± 0.0^a^
*Lepista nuda *	2.10 ± 0.1^a^	40.61 ± 2.7^i^	10.19 ± 1.5^b^	2.10 ± 0.0^c^	ND	ND
*Lentinus cladopus *	9.21 ± 0.5^f^	61.19 ± 3.0^n^	21.18 ± 1.3^j^	3.32 ± 0.2^d^	0.6 ± 0.0^b^	0.05 ± 0.0^a^
*Pleurotus cystidiosus *	10.79 ± 0.6^g^	59.11 ± 2.7^m^	19.12 ± 1.5^i^	4.10 ± 0.6^e^	0.6 ± 0.0^b^	0.04 ± 0.0^a^
*Russula lepida *	5.15 ± 0.1^d^	32.61 ± 3.6^f^	13.16 ± 1.2^e^	2.31 ± 0.1^c^	ND	ND
*R. mairei *	4.26 ± 0.1^c^	21.60 ± 2.1^a^	12.81 ± 1.6^d^	2.12 ± 0.0^c^	ND	ND

ND = not detected.

Values are expressed as mean ± SE and different letters represent the significant difference in each column with *p* ≤ 0.05 according to Tukey's test.

**Table 4:** Percent fatty acid composition of all the twenty wild culinary mushroom species collected from different regions of northern Himalayas.

Species	C9:00	C10:0	C12:0	C16:0	C161:1	C17:1	C18:1	C18:2	C20:2	UI
*Agaricus arvensis *	0.06 ± 0.0^a^	0.30 ± 0.01^a^	0.28 ± 0.0^a^	1.86 ± 0.03^a^	0.06 ± 0.0^a^	0.09 ± 0.0^a^	0.18 ± 0.0^a^	3.24 ± 0.0^a^	2.21 ± 0.2^a^	0.57 ± 0.02^a^
*A. campestris *	0.03 ± 0.0^a^	0.26 ± 0.02^a^	0.21 ± 0.0^a^	1.19 ± 0.02^a^	0.09 ± 0.0^a^	0.02 ± 0.0^a^	0.35 ± 0.0^a^	2.04 ± 0.0^a^	2.18 ± 0.3^a^	0.46 ± 0.02^a^
*A. comtulus *	0.06 ± 0.0^a^	0.22 ± 0.0^a^	ND	1.17 ± 0.01^a^	0.07 ± 0.0^a^	0.09 ± 0.0^a^	0.38 ± 0.0^a^	1.73 ± 0.0^a^	1.34 ± 0.1^a^	0.36 ± 0.01^a^
*A. silvicola *	0.08 ± 0.0^a^	0.33 ± 0.0^a^	0.22 ± 0.0^a^	1.56 ± 0.02^a^	0.09 ± 0.0^a^	0.01 ± 0.0^a^	0.15 ± 0.0^a^	1.54 ± 0.0^a^	2.21 ± 0.3^a^	0.40 ± 0.01^a^
*Amanita caesarea *	0.04 ± 0.0^a^	0.32 ± 0.02^a^	0.19 ± 0.0^a^	1.96 ± 0.02^a^	0.12 ± 0.0^a^	0.05 ± 0.0^a^	0.31 ± 0.0^a^	2.19 ± 0.0^a^	1.28 ± 0.0^a^	0.39 ± 0.01^a^
*A. citrina *	0.06 ± 0.0^a^	0.28 ± 0.01^a^	ND	0.91 ± 0.0^a^	0.11 ± 0.0^a^	0.08 ± 0.0^a^	0.31 ± 0.0^a^	1.59 ± 0.0^a^	1.68 ± 0.0^a^	0.37 ± 0.01^a^
*A. fulva *	0.04 ± 0.0^a^	0.21 ± 0.01^a^	0.25 ± 0.02^a^	0.10 ± 0.0^a^	0.08 ± 0.0^a^	0.04 ± 0.0^a^	0.15 ± 0.0^a^	1.64 ± 0.0^a^	1.23 ± 0.0^a^	0.31 ± 0.02^a^
*Cantharellus cibarius *	0.07 ± 0.0^a^	0.33 ± 0.02^a^	0.22 ± 0.01^a^	1.10 ± 0.02^a^	0.07 ± 0.0^a^	0.01 ± 0.0^a^	0.37 ± 0.0^a^	2.94 ± 0.0^a^	1.18 ± 0.1^a^	0.45 ± 0.02^a^
*Conocybe tenera *	0.06 ± 0.0^a^	0.20 ± 0.0^a^	ND	0.07 ± 0.0^a^	0.05 ± 0.0^a^	0.02 ± 0.0^a^	0.35 ± 0.0^a^	0.14 ± 0.0^a^	1.38 ± 0.0^a^	0.19 ± 0.0^a^
*Gymnopilus junonius *	0.05 ± 0.0^a^	0.31 ± 0.01^a^	0.14 ± 0.01^a^	1.87 ± 0.03^a^	0.08 ± 0.0^a^	0.02 ± 0.0^a^	0.88 ± 0.0^a^	0.49 ± 0.0^a^	1.21 ± 0.1^a^	0.26 ± 0.01^a^
*Hygrocybe coccinea *	0.04 ± 0.0^a^	0.25 ± 0.02^a^	0.17 ± 0.0^a^	0.33 ± 0.0^a^	0.01 ± 0.0^a^	0.05 ± 0.0^a^	0.17 ± 0.0^a^	0.24 ± 0.0^a^	1.19 ± 0.0^a^	0.16 ± 0.0^a^
*H. nivea *	0.07 ± 0.0^a^	0.22 ± 0.01^a^	ND	0.05 ± 0.0^a^	0.04 ± 0.0^a^	0.01 ± 0.0^a^	0.53 ± 0.0^a^	0.04 ± 0.0^a^	1.38 ± 0.2^a^	0.20 ± 0.0^a^
*Inocybe splendens *	0.02 ± 0.0^a^	0.19 ± 0.01^a^	0.23 ± 0.01^a^	0.89 ± 0.0^a^	0.02 ± 0.0^a^	0.03 ± 0.0^a^	0.18 ± 0.0^a^	0.10 ± 0.0^a^	0.20 ± 0.0^a^	0.05 ± 0.0^a^
*Lactarius pubescens *	0.01 ± 0.0^a^	0.23 ± 0.01^a^	0.16 ± 0.0^a^	0.75 ± 0.0^a^	0.07 ± 0.0^a^	0.08 ± 0.0^a^	0.26 ± 0.0^a^	3.44 ± 0.0^a^	0.83 ± 0.0^a^	0.46 ± 0.02^a^
*Laccaria laccata *	0.02 ± 0.0^a^	0.22 ± 0.0^a^	ND	1.01 ± 0.0^a^	0.06 ± 0.0^a^	0.09 ± 0.0^a^	0.92 ± 0.0^a^	2.84 ± 0.0^a^	0.29 ± 0.0^a^	0.42 ± 0.01^a^
*Lepista nuda *	0.03 ± 0.0^a^	0.24 ± 0.0^a^	ND	0.43 ± 0.0^a^	0.01 ± 0.0^a^	0.02 ± 0.0^a^	0.20 ± 0.0^a^	2.04 ± 0.0^a^	0.37 ± 0.0^a^	0.26 ± 0.0^a^
*Lentinus cladopus *	0.09 ± 0.0^a^	0.33 ± 0.02^a^	0.32 ± 0.0^a^	1.46 ± 0.0^a^	0.55 ± 0.0^a^	0.01 ± 0.0^a^	0.32 ± 0.0^a^	2.45 ± 0.0^a^	2.95 ± 0.0^a^	0.57 ± 0.02^a^
*Pleurotus cystidiosus *	0.06 ± 0.0^a^	0.31 ± 0.01^a^	0.28 ± 0.0^a^	1.35 ± 0.0^a^	0.09 ± 0.0^a^	0.05 ± 0.0^a^	0.98 ± 0.0^a^	2.04 ± 0.0^a^	2.21 ± 0.2^a^	0.52 ± 0.02^a^
*Russula lepida *	0.06 ± 0.0^a^	0.19 ± 0.01^a^	ND	0.33 ± 0.0^a^	0.05 ± 0.0^a^	0.05 ± 0.0^a^	0.49 ± 0.0^a^	2.04 ± 0.3^a^	1.65 ± 0.0^a^	0.42 ± 0.01^a^
*R. mairei *	0.01 ± 0.0^a^	0.17 ± 0.01^a^	ND	0.26 ± 0.0^a^	0.08 ± 0.0^a^	0.08 ± 0.0^a^	0.63 ± 0.0^a^	2.01 ± 0.2^a^	0.10 ± 0.0^a^	0.28 ± 0.01^a^

ND = not detected.

Values are expressed as mean ± SE and letters in the superscripts represent the significant difference in each column with *p* ≤ 0.05 according to Tukey's test.

**Table 5:** Percent amino acids composition of all the wild culinary species collected from northern Himalayas.

Species	Aspartic acid	Arginine	Alanine	Proline	Tyrosine
*Agaricus arvensis *	0.38 ± 0.0^a^	0.27 ± 0.0^a^	0.14 ± 0.0^a^	0.06 ± 0.0^a^	0.19 ± 0.0^a^
*A. campestris *	0.30 ± 0.0^a^	0.19 ± 0.0^a^	0.10 ± 0.0^a^	0.02 ± 0.0^a^	0.14 ± 0.0^a^
*A*. *comtulus *	0.27 ± 0.0^a^	0.15 ± 0.0^a^	0.10 ± 0.0^a^	0.02 ± 0.0^a^	0.12 ± 0.0^a^
*A. silvicola *	0.29 ± 0.0^a^	0.23 ± 0.0^a^	0.08 ± 0.0^a^	0.03 ± 0.0^a^	0.17 ± 0.0^a^
*Amanita caesarea *	0.39 ± 0.0^a^	0.25 ± 0.0^a^	0.13 ± 0.0^a^	0.07 ± 0.0^a^	0.21 ± 0.0^a^
*A. citrina *	0.21 ± 0.0^a^	0.20 ± 0.0^a^	0.11 ± 0.0^a^	0.04 ± 0.0^a^	0.15 ± 0.0^a^
*A. fulva *	0.33 ± 0.0^a^	0.17 ± 0.0^a^	0.07 ± 0.0^a^	0.03 ± 0.0^a^	0.16 ± 0.0^a^
*Cantharellus cibarius *	0.20 ± 0.0^a^	0.29 ± 0.0^a^	0.13 ± 0.0^a^	0.06 ± 0.0^a^	0.20 ± 0.0^a^
*Conocybe tenera *	0.31 ± 0.0^a^	0.21 ± 0.0^a^	0.06 ± 0.0^a^	0.01 ± 0.0^a^	0.10 ± 0.0^a^
*Gymnopilus junonius *	0.25 ± 0.0^a^	0.25 ± 0.0^a^	0.14 ± 0.0^a^	0.05 ± 0.0^a^	0.17 ± 0.0^a^
*Hygrocybe coccinea *	0.22 ± 0.0^a^	0.12 ± 0.0^a^	0.04 ± 0.0^a^	0.01 ± 0.0^a^	0.11 ± 0.0^a^
*H. nivea *	0.19 ± 0.0^a^	0.13 ± 0.0^a^	0.04 ± 0.0^a^	0.01 ± 0.0^a^	0.11 ± 0.0^a^
*Inocybe splendens *	0.23 ± 0.0^a^	0.20 ± 0.0^a^	0.08 ± 0.0^a^	0.04 ± 0.0^a^	0.14 ± 0.0^a^
*Lactarius pubescens *	0.22 ± 0.0^a^	0.23 ± 0.0^a^	0.10 ± 0.0^a^	0.04 ± 0.0^a^	0.15 ± 0.0^a^
*Laccaria laccata *	0.24 ± 0.0^a^	0.22 ± 0.0^a^	0.08 ± 0.0^a^	0.01 ± 0.0^a^	0.13 ± 0.0^a^
*Lepista nuda *	0.33 ± 0.0^a^	0.21 ± 0.0^a^	0.06 ± 0.0^a^	0.01 ± 0.0^a^	0.12 ± 0.0^a^
*Lentinus cladopus *	0.36 ± 0.0^a^	0.29 ± 0.0^a^	0.13 ± 0.0^a^	0.05 ± 0.0^a^	0.18 ± 0.0^a^
*Pleurotus cystidiosus *	0.35 ± 0.0^a^	0.27 ± 0.0^a^	0.12 ± 0.0^a^	0.04 ± 0.0^a^	0.20 ± 0.0^a^
*Russula lepida *	0.21 ± 0.0^a^	0.20 ± 0.0^a^	0.06 ± 0.0^a^	0.02 ± 0.0^a^	0.12 ± 0.0^a^
*R. mairei *	0.20 ± 0.0^a^	0.19 ± 0.0^a^	0.06 ± 0.0^a^	0.01 ± 0.0^a^	0.11 ± 0.0^a^

Values are expressed as mean ± SE and letters in superscript represent the significant difference in each column with *p* ≤ 0.05 according to Tukey's test.

**Table 6:** Tocopherol composition (*µ*g/g) of twenty species collected from northern Himalayan regions.

Species	*α*-tocopherol	*β*-tocopherol	*γ*-tocopherol	Total
*Agaricus arvensis *	0.65 ± 0.0^a^	1.24 ± 0.0^b^	1.12 ± 0.0^b^	3.01 ± 0.0^d^
*A. campestris *	0.62 ± 0.0^a^	1.20 ± 0.0^b^	1.10 ± 0.0^b^	2.92 ± 0.0^c^
*A*. *comtulus *	0.55 ± 0.0^a^	0.96 ± 0.0^a^	ND	1.51 ± 0.0^b^
*A. silvicola *	0.75 ± 0.0^a^	0.87 ± 0.0^a^	0.98 ± 0.0^a^	2.60 ± 0.0^c^
*Amanita caesarea *	0.95 ± 0.0^a^	1.56 ± 0.0^b^	1.22 ± 0.0^b^	3.73 ± 0.0^d^
*A. citrina *	0.43 ± 0.0^a^	1.16 ± 0.0^b^	ND	1.59 ± 0.0^b^
*A. fulva *	0.41 ± 0.0^a^	1.42 ± 0.0^b^	ND	1.83 ± 0.0^b^
*Cantharellus cibarius *	1.25 ± 0.0^b^	1.79 ± 0.0^b^	1.29 ± 0.0^b^	4.33 ± 0.0^e^
*Conocybe tenera *	0.25 ± 0.0^a^	0.86 ± 0.0^a^	ND	1.11 ± 0.0^b^
*Gymnopilus junonius *	0.83 ± 0.0^a^	1.56 ± 0.0^b^	1.17 ± 0.0^b^	3.56 ± 0.0^d^
*Hygrocybe coccinea *	0.46 ± 0.0^a^	0.72 ± 0.0^a^	ND	1.18 ± 0.0^b^
*H. nivea *	0.41 ± 0.0^a^	0.70 ± 0.0^a^	ND	1.11 ± 0.0^b^
*Inocybe splendens *	0.25 ± 0.0^a^	0.52 ± 0.0^a^	ND	0.77 ± 0.0^a^
*Lactarius pubescens *	0.66 ± 0.0^a^	1.02 ± 0.0^b^	0.92 ± 0.0^a^	2.60 ± 0.0^c^
*Laccaria laccata *	0.35 ± 0.0^a^	1.17 ± 0.0^b^	ND	1.52 ± 0.0^b^
*Lepista nuda *	0.21 ± 0.0^a^	0.93 ± 0.0^a^	ND	1.14 ± 0.0^b^
*Lentinus cladopus *	0.85 ± 0.0^a^	1.51 ± 0.0^b^	1.19 ± 0.0^b^	3.55 ± 0.0^d^
*Pleurotus cystidiosus *	1.15 ± 0.0^b^	1.62 ± 0.0^b^	1.16 ± 0.0^b^	3.93 ± 0.0^d^
*Russula lepida *	0.32 ± 0.0^a^	0.65 ± 0.0^a^	ND	0.97 ± 0.0^a^
*R. mairei *	0.31 ± 0.0^a^	0.59 ± 0.0^a^	ND	0.90 ± 0.0^a^

ND = not detected.

Values are expressed as mean ± SE and different letters represent the significant difference in each column with *p* ≤ 0.05 according to Tukey's test.

**Table 7:** Other bioactive compounds evaluated from all the species.

Species	*β*-carotene (*µ*g/100 g)	Lycopene (*µ*g/100 g)	Phenolic compounds (mg/100 g of gallic acid)	Flavonoids (mg gallic acid equivalents/g)	Ascorbic acid (mg/100 g)	Anthocyanidins (mg cyanidin chloride/100 g extract)
*Agaricus arvensis *	0.75 ± 0.0^a^	0.38 ± 0.0^a^	55.13 ± 2.1^i^	2.70 ± 0.0^b^	0.85 ± 0.0^a^	12.19 ± 0.2^f^
*A. campestris *	0.50 ± 0.0^a^	0.27 ± 0.0^a^	43.17 ± 1.7^h^	2.25 ± 0.0^b^	0.50 ± 0.0^a^	10.26 ± 0.3^d^
*A*. *comtulus *	0.70 ± 0.0^a^	0.30 ± 0.0^a^	31.16 ± 2.1^f^	2.18 ± 0.0^b^	0.57 ± 0.0^a^	9.90 ± 0.5^c^
*A. silvicola *	0.48 ± 0.0^a^	0.27 ± 0.0^a^	39.12 ± 3.1^g^	2.14 ± 0.0^b^	0.48 ± 0.0^a^	11.02 ± 0.4^e^
*Amanita caesarea *	0.71 ± 0.0^a^	0.29 ± 0.0^a^	62.32 ± 2.9^j^	4.17 ± 0.0^c^	0.91 ± 0.0^a^	17.25 ± 0.8^e^
*A. citrina *	0.57 ± 0.0^a^	0.39 ± 0.0^a^	41.13 ± 1.1^h^	3.35 ± 0.0^c^	0.77 ± 0.0^a^	12.93 ± 0.5^f^
*A. fulva *	0.39 ± 0.0^a^	0.21 ± 0.0^a^	39.16 ± 1.8^h^	3.11 ± 0.0^c^	0.79 ± 0.0^a^	13.47 ± 0.6^g^
*Cantharellus cibarius *	0.79 ± 0.0^a^	0.33 ± 0.0^a^	63.36 ± 2.5^j^	4.45 ± 0.0^c^	0.99 ± 0.0^a^	14.25 ± 0.7^h^
*Conocybe tenera *	0.45 ± 0.0^a^	0.20 ± 0.0^a^	35.1 ± 1.4^f^	1.90 ± 0.0^b^	0.35 ± 0.0^a^	7.99 ± 0.4^b^
*Gymnopilus junonius *	0.70 ± 0.0^a^	0.31 ± 0.0^a^	53.17 ± 3.2^i^	3.95 ± 0.0^c^	0.80 ± 0.0^a^	14.12 ± 0.8^h^
*Hygrocybe coccinea *	0.37 ± 0.0^a^	0.25 ± 0.0^a^	30.11 ± 3.1^f^	2.98 ± 0.02^b^	0.37 ± 0.0^a^	10.21 ± 0.3^d^
*H. nivea *	0.38 ± 0.0^a^	0.22 ± 0.0^a^	19.12 ± 2.1^e^	2.14 ± 0.0^b^	0.32 ± 0.0^a^	6.92 ± 0.5^a^
*Inocybe splendens *	0.21 ± 0.0^a^	0.19 ± 0.0^a^	18.32 ± 2.1^e^	2.37 ± 0.0^b^	0.20 ± 0.0^a^	6.39 ± 0.4^a^
*Lactarius pubescens *	0.47 ± 0.0^a^	0.33 ± 0.0^a^	51.19 ± 3.1^i^	3.34 ± 0.0^c^	0.39 ± 0.0^a^	12.59 ± 0.3^f^
*Laccaria laccata *	0.40 ± 0.0^a^	0.30 ± 0.0^a^	39.62 ± 2.8^f^	2.51 ± 0.0^b^	0.35 ± 0.0^a^	12.62 ± 0.6^f^
*Lepista nuda *	0.39 ± 0.0^a^	0.20 ± 0.0^a^	23.37 ± 2.1^f^	2.47 ± 0.0^b^	0.34 ± 0.0^a^	7.95 ± 0.3^b^
*Lentinus cladopus *	0.75 ± 0.0^a^	0.30 ± 0.0^a^	55.13 ± 1.9^i^	3.90 ± 0.0^c^	0.77 ± 0.0^a^	13.72 ± 0.5^e^
*Pleurotus cystidiosus *	0.79 ± 0.0^a^	0.28 ± 0.0^a^	53.20 ± 2.7^i^	3.99 ± 0.0^c^	0.82 ± 0.0^a^	15.25 ± 0.6^e^
*Russula lepida *	0.27 ± 0.0^a^	0.20 ± 0.0^a^	30.76 ± 2.2^f^	1.98 ± 0.0^b^	0.29 ± 0.0^a^	6.24 ± 0.2^d^
*R. mairei *	0.23 ± 0.0^a^	0.23 ± 0.0^a^	27.10 ± 3.1^f^	1.14 ± 0.0^b^	0.27 ± 0.0^a^	6.14 ± 0.2^d^

Values are expressed as mean ± SE and different letters represent the significant difference in each column with *p* ≤ 0.05 according to Tukey's test.

**Table 8:** EC_50_ values for different antioxidant assays on twenty wild culinary species collected from northern Himalayas.

Species	DPPH radical scavenging activity (mg/mL)	ABTS (mg/mL)	Reducing power (mg/mL)	Fe^2+^ chelating activity (mg/mL)	Scavenging on superoxide anion radical (mg/mL)	FRAP (*μ*mol Fe^2+^ equivalents/g DW)
*Agaricus arvensis *	2.12 ± 0.4	3.19 ± 0.3	2.27 ± 0.3	1.14 ± 0.3	1.34 ± 0.1	1.74 ± 0.1
*A. campestris *	3.28 ± 0.5	3.10 ± 0.4	3.12 ± 0.5	2.02 ± 0.2	1.41 ± 0.5	1.56 ± 0.3
*A*. *comtulus *	3.14 ± 0.2	3.27 ± 0.3	3.11 ± 0.6	1.92 ± 0.1	2.04 ± 0.5	1.44 ± 0.1
*A. silvicola *	3.08 ± 0.3	3.35 ± 0.5	3.95 ± 0.2	1.84 ± 0.2	2.09 ± 0.4	1.41 ± 0.0
*Amanita caesarea *	2.02 ± 0.2	1.45 ± 0.6	2.44 ± 0.1	1.40 ± 0.4	0.44 ± 0.3	1.86 ± 0.1
*A. citrina *	3.21 ± 0.4	3.19 ± 0.4	2.57 ± 0.7	2.24 ± 0.3	1.11 ± 0.2	1.50 ± 0.0
*A. fulva *	3.29 ± 0.3	3.25 ± 0.3	2.78 ± 0.2	1.92 ± 0.5	1.10 ± 0.3	1.46 ± 0.2
*Cantharellus cibarius *	1.76 ± 0.2	1.59 ± 0.4	1.62 ± 0.5	1.27 ± 0.2	0.39 ± 0.4	1.85 ± 0.3
*Conocybe tenera *	5.13 ± 0.2	3.21 ± 0.6	5.12 ± 0.3	1.74 ± 0.5	1.09 ± 0.2	1.56 ± 0.0
*Gymnopilus junonius *	2.58 ± 0.2	2.84 ± 0.4	1.95 ± 0.2	2.21 ± 0.3	1.27 ± 0.6	0.78 ± 0.0
*Hygrocybe coccinea *	3.82 ± 0.2	3.29 ± 0.5	3.12 ± 0.8	1.35 ± 0.2	1.98 ± 0.2	1.11 ± 0.0
*H. nivea *	3.64 ± 0.3	4.26 ± 0.5	3.11 ± 0.2	1.45 ± 0.6	1.94 ± 0.1	1.13 ± 0.0
*Inocybe splendens *	4.12 ± 0.3	3.15 ± 0.6	5.19 ± 0.1	1.76 ± 0.4	1.99 ± 0.7	1.24 ± 0.0
*Lactarius pubescens *	2.87 ± 0.2	3.39 ± 0.4	4.87 ± 0.3	1.87 ± 0.2	2.21 ± 0.6	1.62 ± 0.4
*Laccaria laccata *	3.19 ± 0.4	3.23 ± 0.3	4.12 ± 0.2	1.94 ± 0.6	1.91 ± 0.2	1.56 ± 0.0
*Lepista nuda *	3.71 ± 0.3	3.26 ± 0.4	4.21 ± 0.3	2.04 ± 0.5	1.84 ± 0.1	1.36 ± 0.0
*Lentinus cladopus *	2.15 ± 0.5	2.29 ± 0.9	2.86 ± 0.2	2.19 ± 0.1	1.10 ± 0.2	1.66 ± 0.0
*Pleurotus cystidiosus *	2.17 ± 0.6	2.95 ± 0.2	2.57 ± 0.1	2.11 ± 0.3	1.04 ± 0.1	1.58 ± 0.3
*Russula lepida *	3.10 ± 0.2	3.21 ± 0.1	4.10 ± 0.3	2.84 ± 0.2	1.23 ± 0.2	1.19 ± 0.0
*R. mairei *	3.17 ± 0.3	3.11 ± 0.1	5.16 ± 0.2	2.95 ± 0.3	1.14 ± 0.1	2.16 ± 0.0

Values are expressed as mean ± SE.

## Abbreviations

AlCl_3_: Aluminum trichloride
ANOVA: Analysis of variance
D.W.: Distilled water
DPPH: 2,2-Diphenyl-1-picrylhydrazyl
FeCl_3_: Ferric chloride
FRAP: Ferric reducing antioxidant power
g: Grams
GAEs: Gallic acid equivalents
GC: Gas Chromatography
HCl: Hydrochloric acid
H_2_O: Water
H_3_PO_4_: Phosphoric acid
HPLC: High performance liquid chromatography
K_2_HPO_4_: Potassium hydrogen, phosphate
L: Liters
m: Meters
mg: Milligrams
mL: Milliliters
mM: Millimolar
MTBE: Methyl tertiary-butyl ether
MUFA: Monounsaturated fatty acids
N: Nitrogen
NaOH: Sodium hydroxide
ND: Not detected
°C: Degree centigrade
PUFA: Polyunsaturated fatty acids
rpm: Rotation per minute
SFA: Saturated fatty acids
TPTZ: Tripyridyltriazine
vol.: Volume
%: Percent
*μ*g: Microgram
*μ*M: Micromolar
UI: Unsaturation index
UFA: Unsaturated fatty acids
UPLC: Ultra performance liquid chromatography.
